# The Association Between Myocardial Infarction and Loneliness Among Adults in the Saudi Population: A Comparative Analytical Study

**DOI:** 10.1155/crp/6360092

**Published:** 2026-10-23

**Authors:** Hessah Alshammari, Norah Alshafi, Tharaa Alhowaish, Lina Almohsen, Dena Alsuhaibani, Hassan Alabdullatif, Sarah Mazi, Thamir Al-khlaiwi

**Affiliations:** ^1^ Department of Cardiac Sciences College of Medicine King Saud University Riyadh Saudi Arabia ksu.edu.sa; ^2^ Department of Physiology College of Medicine King Saud University Riyadh Saudi Arabia ksu.edu.sa

**Keywords:** cardiovascular disease, myocardial infarction, physical inactivity, Saudi population, smoking, social loneliness

## Abstract

**Background & Aim:**

Cardiovascular disease (CVD) represents a major global health crisis, accounting for a significant fraction of overall morbidity and mortality rates. While growing evidence indicates that poor cardiovascular health may be associated with higher loneliness, studies on this association within the Middle Eastern context, particularly in Saudi Arabia, are limited. Given the unique social and cultural characteristics of Saudi Arabia, this study aimed to examine the association of myocardial infarction (MI) and loneliness among adults in the country.

**Methods:**

This comparative analytical study, using questionnaires, was conducted at King Khaled University Hospital in Riyadh. We recruited 173 Saudi adult patients aged between 18 and over 70 years with a confirmed diagnosis of MI and compared them with 446 Saudi adult healthy participants without CVD. Participants completed the validated Arabic translation of the De Jong Gierveld Loneliness Scale. Sociodemographic, behavioral, clinical, and treatment‐related data were also collected. Bivariate and multivariable logistic regression analyses were used to identify the association between high loneliness and MI (score ≥ 3).

**Results:**

A high loneliness score was reported by 28.4% of all participants. Multivariable analysis identified statistically significant associations between loneliness and the presence of heart disease, younger age, physical inactivity, and smoking (OR = 4.88, 95% CI 2.46–9.67, *p* < 0.001). In cardiac patients, higher physical activity and better diabetes management were correlated with lower loneliness scores, whereas chronic kidney dysfunction significantly increased loneliness scores (*p* = 0.03).

**Conclusions:**

We found that, within the highly culturally and socially oriented population of Saudi Arabia, MI in adults was associated with greater loneliness compared to healthy participants. Loneliness is also significantly associated with smoking, physical inactivity, and comorbidities in MI patients. These findings highlight the need for culturally tailored interventions that integrate psychosocial support into cardiac care, particularly for vulnerable subgroups.

## 1. Introduction

Cardiovascular disease (CVD) represents a major health crisis in the Middle East, accounting for a significant proportion of overall morbidity and mortality rates and for 34% of total fatalities(23)[1, 2]. In the context of the declining mortality rates and disability‐adjusted life years (DALYs), which have surpassed 30% since 1990 [3], CVD incidence continues to increase, particularly in regions classified as low and lower‐middle on the sociodemographic index (SDI) [4]. CVD is also a significant economic challenge, and experts anticipate that its cost will triple to $9.8 billion by 2035. These costs cover expenses associated with treatment, lower productivity, and early death [2]. The prevalence of CVD is influenced by a multitude of contributing factors in the specific context of Saudi Arabia, where approximately 1.6% of all people aged 15 years and older suffer from CVD, and more men (1.9%) are diagnosed with the condition than women (1.4%). While it is more common among people aged ≥ 65 years or older [5], recent studies have shown a high prevalence of CVD in the younger population, driven by multifactorial risks including smoking, diabetes, hypertension, and high cholesterol levels [4]. Astonishingly, a recent study revealed a mortality prevalence of 4% in the young Saudi population (aged ≤ 50 years), which is similar to the prevalence in older patients [6]. The incidence rate is exacerbated by sedentary lifestyles and rising obesity rates [7]. Recent research has increasingly focused on the association of CVD and social interaction and cultural status, with an increasing number of researchers acknowledging that the condition may lead to loneliness [8]. Incorporating social determinants, such as loneliness, into analyses provides a more comprehensive perspective, and addressing these correlates may lead to improved prevention strategies and population health outcomes.

Loneliness is the subjective feeling of being alone, disconnected, or unsatisfied with one’s social relationships. It is well‐established that loneliness is influenced by factors such as age, gender, marital status, education, socioeconomic status, living situation, and cultural and environmental characteristics [9]. Recently, a growing body of evidence has indicated that loneliness is associated with various health‐related issues, particularly those affecting physical well‐being. Studies have shown that individuals with high loneliness scores are at a significantly higher risk of developing physical health issues, such as poorer patient outcomes, greater inflammation, and reduced immune function, increasing their risk of early mortality [8, 10, 11]. Significantly, loneliness is associated with increased CVD mortality, with older individuals experiencing up to a 29% higher risk of CVD due to loneliness [12].

Sociocultural characteristics play a vital role in shaping social connections in Saudi Arabia. A qualitative study demonstrated that sociocultural norms, religious beliefs, and family values significantly influence how Saudi patients with cardiac disease engage in lifestyle changes after experiencing cardiac events [13]. Recent demographic shifts, such as a decline in extended families, an increase in single‐person households, and growing urbanization, have weakened traditional support structures, raising concerns regarding loneliness and reduced social cohesion in Saudi society [14]. There is a notable scarcity of regional studies in Saudi Arabia assessing the association of CVD with loneliness, leaving a significant gap in our understanding of this association. This lack of research underscores the importance of culturally relevant data for effective future public health planning and the development of interventions that reflect the unique social and cultural dynamics of the Saudi population. Without such data, health strategies may overlook key psychosocial determinants and fail to address patients’ needs. To address this gap, our study explored the association of myocardial infarction (MI) with loneliness among patients in Saudi Arabia.

## 2. Methods

### 2.1. Study Design and Setting

This questionnaire‐based study employed a cross‐sectional comparative analytical design and was conducted at King Khaled University Hospital, Riyadh, Saudi Arabia, between May 01, 2024, and June 30, 2024. The study adhered to the principles of the Declaration of Helsinki and was approved by the Institutional Review Board of the College of Medicine, King Saud University (approval number: Research Project No. E‐24‐8857).

### 2.2. Participants

The sample comprised 619 participants: 173 MI patients and 446 healthy participants. The study group (MI patients) was recruited using a convenience sampling technique from patients diagnosed with MI at King Fahad Cardiac Center. All participants provided consent to participate. The inclusion criteria were as follows. Participants had to be Saudi citizens living in Saudi Arabia, aged 18–over 70 years, and diagnosed with MI by angiographic findings (indicating occlusion of the coronary artery with impact on the cardiac muscle), ECG, and echo findings by the cardiologist, and admitted to the cardiac center. Patients without Saudi nationality and those living outside of Saudi Arabia were excluded. The control group (healthy participants) was recruited from blood bank donors and hospital employees and adults with no self‐reported history of MI or diagnosed CVD.

The sample size was calculated using the single proportion equation as follows: *n = Z*2 x *P* (1*−P*)/*d*2, where *Z* = 1.96 (95% confidence level), *d* = 0.05, and *P* = the proportion of subjects at risk, which was assumed to be 1.6% [3]:

(1) 1.962×0.01610.016−0.052=394.

While the calculated sample size was 394 patients, we only recruited 173.

### 2.3. Variables

We used the De Jong Gierveld Scale, a validated measure of loneliness, to assess overall, emotional, and social loneliness [15]. The authors have previously used a validated Arabic translation, and a pilot study was conducted as a foundation for the study [16]. A score of ≥ 3 was used to indicate relatively high loneliness levels, consistent with previous studies utilizing the De Jong Gierveld Loneliness Scale, and to facilitate binary logistic regression analysis [6]. In addition, its relatively brief structure made it suitable for the current questionnaire‐based study. Sociodemographic, behavioral, and clinical variables were assessed using structured questionnaires and, when available, verified against medical records. Information about age, sex, educational level, marital status, employment, and income was self‐reported. Smoking status was categorized as current, former, or never smoker. Clinical variables, including diabetes mellitus, hypertension, dyslipidemia, and history of MI, were extracted from hospital electronic records. Medication adherence and treatment variables (beta‐blockers, statins, and antiplatelets) were also included as covariates in the multivariable model and verified by the medical record. Covariates included in the multivariable models were selected a priori based on previous literature and their potential role as confounders of the association between MI and loneliness. These variables included age, sex, marital status, education, employment status, smoking, physical activity, body mass index, and comorbidities. Because of the cross‐sectional design, temporal relationships could not be established; therefore, the analyses were intended to adjust for potential confounding rather than distinguish confounding from mediation. For the healthy participants, the De Jong Gierveld Scale questionnaire was distributed via WhatsApp or through personal communications. Personal information, including sociodemographic characteristics, such as age, sex, educational status, occupation, BMI, marital status, behavioral factors, MI history, and comorbidities, was collected from participants’ medical files.

### 2.4. Statistical Analysis

Statistical data analysis: Mean and standard deviation were used to describe continuous measured variables, whereas median and interquartile range (IQR) statistics were used to describe continuous measured variables that violated normality assumptions. Frequencies and percentages were used to describe the categorically measured variables. Multiple‐response dichotomies were applied to describe the questions measured with more than one option (e.g., comorbidity). The Kolmogorov–Smirnov statistical test of normality and skewness and kurtosis tests with histograms were used to assess the statistical normality assumption for metric variables. The variance inflation factor (VIF) and tolerance (Ti) indices were used to evaluate collinearity in metric variables. Cronbach’s alpha was used to determine the internal consistency of the questionnaires. The bivariate chi‐square (*χ* ^2^) test was used to assess the association between categorical variables. The likelihood ratio and continuity corrections were applied to the chi‐squared test tables that did not meet the chi‐squared test’s statistical assumptions (i.e., where cells had counts significantly lower than expected). Multivariable logistic regression was applied to the odds of loneliness and MI. The association between independent predictor variables and analyzed outcomes in the multivariable logistic regression analysis was expressed as odds ratios (ORs) with their associated 95% confidence intervals. Statistical significance was determined at an alpha level of 0.05. A licensed copy of the SPSS IBM statistical computing program, Version 30, was used for statistical data analysis. Finally, this was not a matched case–control study; therefore, no formal matching procedures were applied between the MI and control groups. However, demographic, behavioral, and clinical variables were collected for both groups and were statistically adjusted for in the multivariable analyses to minimize potential confounding effects. Multivariable logistic regression was selected because the primary study outcomes were binary variables [17], making this method suitable for evaluating independent associations while adjusting for potential confounding variables in a comparative cross‐sectional design [14].

## 3. Results

A total of 624 individuals residing in Saudi Arabia were recruited and completed the study questionnaire. The sample included individuals with a prior diagnosis of cardiac disease (*n* = 221) and healthy adults (*n* = 403). The majority of participants were male (64.6%), with 35.4% female. Most participants were aged between 51 and 70 years (43.6%), followed by those aged 30–50 years (33.3%). Younger individuals aged < 30 years (14.5%) and older individuals aged > 70 years (8.6%) accounted for a smaller proportion of the sample. In terms of BMI, a substantial proportion were either overweight (38%) or obese (36.8% combined), while only 24.6% had a normal BMI. Most participants were married (76.7%), and almost half held a university degree (49.1%), with an additional 17.6% reporting postgraduate education. Regarding employment status, over half of the participants were employed full‐time (53.6%), and 42.5% were unemployed. Nearly half of the respondents (49.3%) reported an intermediate household income, with the remainder classified as having low (26%) and high (24.7%) income levels. Smoking was relatively uncommon, with 69.6% of the patients identified as nonsmokers, and alcohol consumption was minimal (1%). Physical activity levels were generally low, with 42.8% of the participants reporting inactivity. Finally, self‐rated loneliness levels, measured using the DeJong Gierveld Loneliness Scale, showed that over half of the participants reported very low to low loneliness (55.6%), with a notable proportion (28.4%) reporting moderate to extreme levels of loneliness, suggesting that loneliness may be a relevant concern in this population (Supporting Table S1). Participants’ medical and cardiac histories revealed that approximately a quarter of them (24.1%) reported a prior MI. Among these, 7.8% had experienced MI within the last 1–2 years, 9.7% between 3 and 4 years ago, and 6.6% five or more years prior (Supporting Table S2).

Regarding ischemic heart disease (IHD), 16.5% had ST‐segment elevation myocardial infarction (STEMI), 4.4% had non‐ST–elevation MI (NSTEMI), 1.9% had stable angina, and 1.3% had unstable angina. Most patients (75.9%) were unaffected by IHD. The number of reported MIs per individual varied: 18.6% reported one MI, 4% reported two, and 1.5% reported three. Recurrent cardiac events and complications such as dysrhythmias or reduced ejection fraction were relatively uncommon, affecting 4% of the sample. Comorbidities were highly prevalent, with dyslipidemia (66.3%) and diabetes (65.7%) being the most common, followed by hypertension (47%). Additional conditions included chronic kidney dysfunction (8.6%), CVD (8.4%), peripheral artery disease (7.5%), chronic obstructive pulmonary disease (COPD; 5.8%), and other less frequent conditions (Figure 1). Statins (80.8%) were the most widely prescribed medication, followed by hypoglycemic agents (53.9%), antiplatelets (53.6%), ACE inhibitors (42%), and beta‐blockers (41%). The use of other medications, such as diuretics, anticoagulants, and antidepressants, was less common, and opioids and benzodiazepines were rarely used (Figure 2).

**FIGURE 1:**
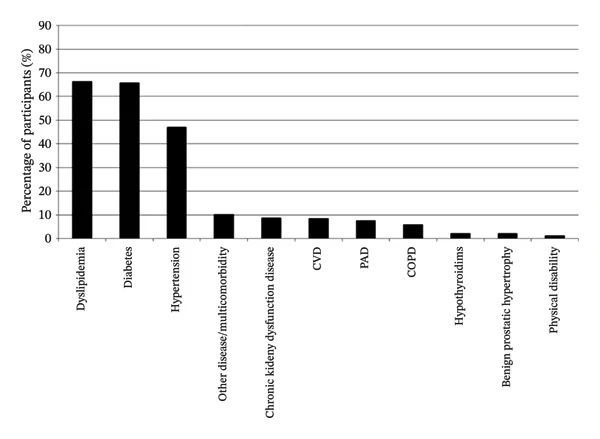
Descriptive analysis of participants’ comorbidities.

**FIGURE 2:**
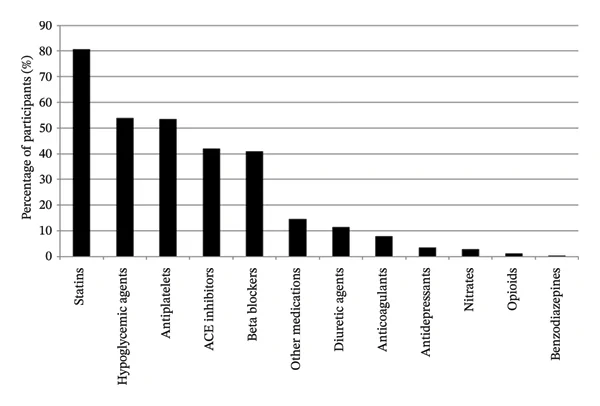
Regularly used medications among participants (regardless of cardiac disease).

Bivariate analysis was conducted to assess factors associated with a higher likelihood of high loneliness levels, defined as a DeJong Loneliness Scale score ≥ 3 (Supporting Table S3A). Educational level, employment status, household income, and smoking behavior were significantly associated with high loneliness scores. Individuals with less than a high school education were significantly more likely to be associated with a high loneliness score than those with greater educational attainment (*χ* ^2^ (1) = 13.84, *p* = 0.003). Similarly, unemployment was associated with a greater likelihood of high loneliness levels (*χ* ^2^ (1) = 13.4, *p* = 0.001). Household income also exhibited a significant relationship, with low‐income individuals being more likely to report a high loneliness score (*χ* ^2^ (2) = 7.12, *p* = 0.028). Smoking status was a significant association, with smokers being more likely to experience greater loneliness than nonsmokers (*χ* ^2^ (1) = 12.21, *p* < 0.001). A more detailed analysis of smoking levels revealed a dose–response relationship, with smoking ≥ 20 cigarettes per day being associated with higher loneliness scores (*χ* ^2^ (3) = 19.26, *p* < 0.001). Other factors, including sex, age group, marital status, alcohol consumption, physical activity level, and BMI, were not significantly associated with high loneliness scores in this sample (all *p* > 0.05).

Bivariate analysis revealed significant associations between cardiac history and the likelihood of high loneliness (DeJong score ≥ 3) (Supporting Table S3B). Individuals with a history of MI were significantly more likely to report a high loneliness score than those without MI (*χ* ^2^ (1) = 69.4, *p* < 0.001). A gradient relationship was observed between the time since the last MI and high loneliness scores, with those experiencing MI more recently at a higher risk (*χ* ^2^ (3) = 71.1, *p* < 0.001). The type of MI was also significantly associated with high loneliness scores, particularly spontaneous MI (26% of highly lonely score individuals) and MI secondary to ischemia (16.4%), *χ* ^2^ (6) = 72.64, *p* < 0.001. Similarly, the presence of IHD, including STEMI, NSTEMI, and stable or unstable angina, was significantly associated with a high loneliness score (*χ* ^2^ (4) = 66.98, *p* < 0.001). The number of MIs experienced also followed a dose–response pattern, with those reporting multiple MIs being more likely to score highly on the loneliness scale (*χ* ^2^ (3) = 65.7, *p* < 0.001). Recurrent cardiac complications, such as dysrhythmias or low ejection fraction, were strongly associated with high loneliness score (*χ* ^2^ (1) = 122.83, *p* < 0.001). Hypertension (*p* < 0.001), multiple comorbidities (*p* < 0.001), chronic kidney disease (CKD) (*p* = 0.023), CVD (*p* = 0.023), hypothyroidism (*p* = 0.034), and benign prostatic hypertrophy (*p* < 0.001) were significantly associated with a higher loneliness score. In contrast, dyslipidemia, diabetes, COPD, and physical disability did not show significant associations. Finally, the use of cardiac‐related medications, including statins, antiplatelets, ACE inhibitors, beta‐blockers, diuretics, anticoagulants, and others, was significantly more common among those with a high loneliness score (all *p* < 0.01), suggesting that greater disease burden and treatment complexity may contribute to the experience of loneliness in this population.

A multivariable binary logistic regression analysis was performed to identify independent factors associated with high loneliness, defined as a DeJong Gierveld Loneliness Scale score ≥ 3 (Table 1). In the multivariable logistic regression analysis, MI remained independently associated with higher loneliness scores after adjustment for demographic, behavioral, and clinical covariates (OR = 4.88, 95% CI: 2.46–9.67, *p* < 0.001). Smokers and physically inactive individuals also demonstrated significantly increased odds of high loneliness. The final model is the result of an iterative process that involved testing several competing models to ensure both statistical robustness and parsimony. All the relevant variables identified in the bivariate analysis were initially considered. Variables that demonstrated redundancy, conceptual overlap, or multicollinearity were systematically excluded to avoid overfitting. Associated factors with consistently high *p*‐values and weak contributions to the model were removed, resulting in a refined best‐fit model that retained only the most relevant associated factors. After adjusting for potential confounders, several factors remained significantly associated with high loneliness scores. Participants aged 50 years or younger had nearly twice the odds of scoring high on loneliness compared with older adults (OR = 1.99, 95% CI [1.16, 3.44], *p* = 0.013). Similarly, individuals with low or inactive physical activity levels were significantly more likely to report high loneliness (OR = 1.55, 95% CI [1.03, 2.36], *p* = 0.038). The presence of heart disease was the strongest associated factor in the model and was associated with nearly five times the odds of a high loneliness score (OR = 4.88, 95% CI [2.46, 9.67], *p* < 0.001). Smoking was also independently associated with increased odds of a high score (OR = 1.84, 95% CI [1.20, 2.83], *p* = 0.005), as well as having other diseases or comorbid conditions (OR = 3.53, 95% CI [1.59, 7.83], *p* = 0.002).

**TABLE 1:** Multivariable binary logistic regression of odds of experiencing high loneliness (loneliness score ≥ 3).

	Multivariate adjusted odds ratio (OR)	95% CI for OR		
		Lower	Upper	*p*‐value
Gender	0.89	0.56	1.43	0.655
Age ≤ 50 years	1.99	1.15	3.43	0.013
Marital state	0.80	0.60	1.06	0.127
Educational level	1.11	0.85	1.45	0.444
Employment state	0.95	0.75	1.20	0.69
Household monthly income [18]	0.85	0.62	1.17	0.333
Body mass index level	0.91	0.76	1.10	0.359
Physical activity level = inactive or very low	1.55	1.02	2.35	0.038
Heart disease	4.88	2.46	9.66	
Smoking status	1.84	1.19	2.83	0.005
ACE inhibitors	1.66	0.95	2.91	0.073
Chronic kidney dysfunction	1.75	0.73	4.2	0.208
Other disease/multicomorbidity	3.53	1.59	7.83	0.002
Number of recurrent MIs ≥ 2	0.65	0.28	1.50	0.32
Constant	0.16			0.002
Dependent variable: Having a high DeJong Gierveld Loneliness Scale score (≥ 3 points) *no*/*yes*.				

To better understand the specific factors contributing to high loneliness scores among individuals with cardiac disease, a subset multivariate binary logistic regression analysis was conducted on 150 participants diagnosed with a cardiac condition (Table 2). The final model was refined through an iterative process in which noncontributing and redundant factors were excluded, yielding a parsimonious model that highlighted the most relevant factors for this subgroup. The analysis revealed that obesity was significantly associated with lower odds of reporting a high loneliness score among patients with cardiac disease (OR = 0.69, 95% CI [0.49, 0.98], *p* = 0.035), suggesting that non‐obese individuals within this group may be at a greater risk for loneliness, as shown in Figures 3 and 4. Similarly, higher levels of physical activity were associated with lower odds of reporting a high loneliness score (OR = 0.45, 95% CI [0.22, 0.93], *p* = 0.031).

**TABLE 2:** Multivariable binary logistic regression analysis of odds of having a high loneliness score among individuals with cardiac disease (*N* = 150).

	Multivariate adjusted odds ratio (OR)	95% CI for OR		
		Lower	Upper	*p*‐value
Age group	0.45	0.20	1.02	0.057
Gender	1.12	0.42	2.98	0.808
Smoking status	1.07	0.48	2.37	0.867
Household monthly income	0.55	0.27	1.12	0.103
Body mass index = obese vs. non‐obese	0.68	0.48	0.97	0.035
Physical activity level	0.44	0.21	0.93	0.031
Comorbidity: chronic kidney dysfunction	6.56	1.19	36.04	0.03
Uptake of beta blockers	0.34	0.12	1.00	0.051
Uptake of ACE inhibitors	1.88	0.82	4.32	0.132
Uptake of statin medications	3.28	0.87	12.35	0.079
Hypoglycemic agents	0.35	0.15	0.80	0.012
Constant	116.12			0.016
Dependent outcome variable: High loneliness score ≥ 3 points: *no*/*yes*				

**FIGURE 3:**
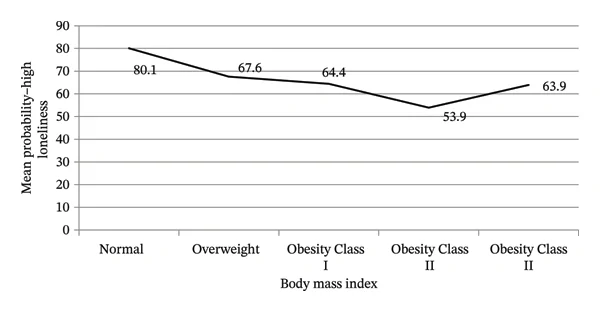
Mean predicted probability of high loneliness according to body mass index (BMI) category among cardiac patients.

**FIGURE 4:**
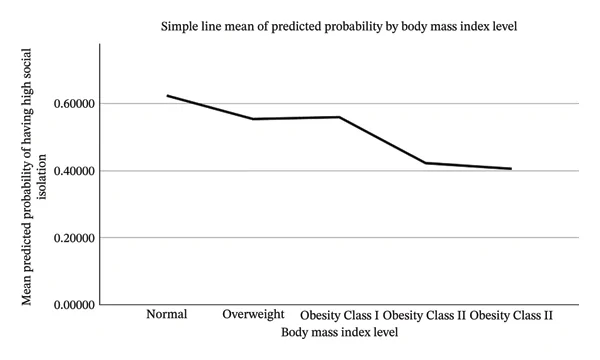
Association between body mass index (BMI) category and the mean predicted probability of high loneliness among cardiac patients.

In contrast, chronic kidney dysfunction was associated with higher odds of a high loneliness score in this subgroup (OR = 6.57, 95% CI [1.20, 36.04], *p* = 0.030). However, the wide confidence interval indicates substantial imprecision in the estimated magnitude of this association, and the finding should therefore be interpreted cautiously. The use of hypoglycemic agents (typically reflecting the presence of diabetes) was associated with significantly reduced odds of high loneliness (OR = 0.36, 95% CI [0.16, 0.80], *p* = 0.012), suggesting a possible link between ongoing diabetes management and improved loneliness. However, this association may also reflect selection or confounding effects. These findings emphasize that, within the cardiac population, factors such as comorbid kidney dysfunction play key roles in increasing loneliness, while obesity, physical activity, and diabetes management are associated with lower odds of loneliness. Given the subgroup sample size and the uncertainty of some estimates, these findings should be interpreted as exploratory and hypothesis‐generating.

A multivariable binary logistic regression model was used as a secondary analysis to identify independent factors of MI, with a specific focus on sociodemographic factors, health behaviors, comorbidities, and loneliness (Table 3). The final model was developed through an iterative process during which several competing models were tested to ensure parsimony, retaining only the most statistically and conceptually relevant factors. The results indicated that females had lower odds of experiencing an MI than males (OR = 0.48, 95% CI [0.23, 0.98], *p* = 0.045). Older age (≥ 51 years) was a strong independent factor, with individuals in this age group being three times more likely to have experienced an MI (OR, 3.02 [95% CI 1.80–5.08], *p* < 0.001) (Figures 5 and 6). Marital status also showed a significant association, with married individuals being more likely to have had an MI than those who were never married (OR 2.10, 95% CI [1.12, 3.93], *p* = 0.021). Employment status was also a significant factor, with unemployed or part‐time employed individuals showing higher odds of MI (OR 2.44, 95% CI [1.26, 4.75], *p* = 0.009). Lower household income (moderate or low vs. high) was among the strongest factors of MI (OR = 7.53, 95% CI [3.15, 17.99], *p* < 0.001). Hypertension (OR = 5.30, 95% CI [2.79, 10.05], *p* < 0.001) and diabetes mellitus (OR = 3.48, 95% CI [1.82, 6.66], *p* < 0.001) were both strongly associated with a history of MI. Interestingly, a positive history of COPD was inversely associated with MI (OR = 0.07, 95% CI [0.005, 0.82], *p* = 0.035).

**TABLE 3:** Multivariable binary logistic regression analysis of the odds of myocardial infarction (MI).

	95% CI for OR			
	Multivariate adjusted odds ratio (OR)	Lower	Upper	*p*‐value
Gender = female	0.47	0.23	0.98	0.045
Age group: ≥ 51 years	3.02	1.80	5.07	< 0.001
Body mass index	0.76	0.57	1.01	0.059
Marital state = ever married vs. never married	2.09	1.11	3.93	0.021
Educational level	1.02	0.71	1.48	0.891
Employment state: unemployed/part‐time job	2.44	1.25	4.74	0.009
Households’ monthly income: moderate or less vs. high	7.52	3.15	17.9	< 0.001
Physical activity level	1.05	0.69	1.60	0.8
Positive medical history of hypertension	5.29	2.79	10.05	< 0.001
Positive medical history of diabetes mellitus	3.48	1.82	6.65	< 0.001
Positive medical history of COPD	0.06	0.00	0.82	0.035
Positive medical history of CVD	2.47	0.75	8.18	0.137
Mean self‐rated loneliness score	1.79	1.49	2.14	< 0.001
Smoking status = smoker	1.98	1.05	3.73	0.034
Constant				< 0.001
Dependent outcome variable: previous myocardial infarction: *no*/*yes*				

**FIGURE 5:**
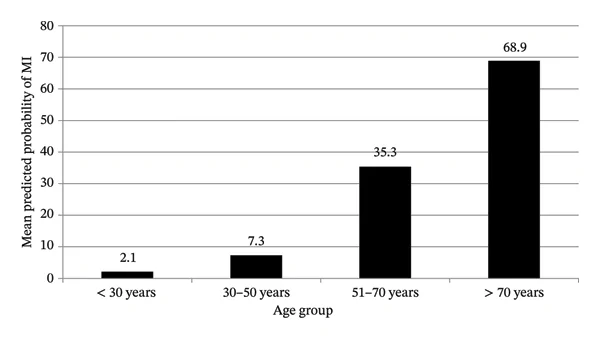
The association between age group and risk of having prior MI.

**FIGURE 6:**
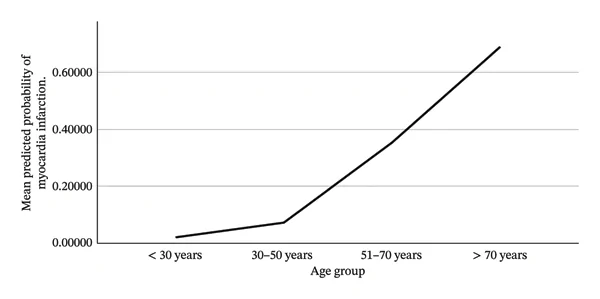
Simple line mean of probability of myocardial infarction by age group.

Notably, the mean self‐rated loneliness score emerged as a significant independent factor of MI—higher loneliness scores were associated with increased odds of having had an MI (OR = 1.80, 95% CI [1.50, 2.15], *p* < 0.001): For each additional one‐point rise in people’s self‐rated loneliness score, their odds of having an MI tended to rise by 79.5% on average. Smoking also independently increased the odds of an MI (OR 1.99, 95% CI [1.05, 3.74], *p* = 0.034). These findings highlight the complex interplay between social, behavioral, and medical factors that contribute to MI risk. Notably, loneliness, along with established clinical risk factors such as hypertension, diabetes, and smoking, independently predicted the odds of having experienced MI.

## 4. Discussion

This study, conducted within Saudi Arabia’s unique and distinguished cultural and social structure, revealed a notable correlation between CVD, particularly MI, and loneliness, as measured by the DeJong Gierveld Loneliness Scale, in a diverse sample. Although the scale allows differentiation between emotional and social loneliness, the present study focused on overall loneliness severity; therefore, the total score was used for analysis. The results underscore the robust association between MI and loneliness. Additionally, elevated loneliness scores were independently associated with younger age, smoking, sedentary lifestyle, and the presence of comorbidities. The findings further suggest that loneliness is strongly intertwined with behavioral clustering. Several studies have shown that CVD is correlated with loneliness, which has been added to other traditional CVD factors [7]. For example, a U.S. cohort study revealed an association between CVD and loneliness in postmenopausal women [19]. However, loneliness in the Middle East is also affected by cultural and social structures.

Our findings indicate that loneliness extends beyond psychological concerns and could be an outcome of cardiovascular morbidity. Higher DeJong loneliness scores were independently associated with increased odds of MI (OR = 1.80, *p* < 0.001). This is consistent with previous studies showing that loneliness is associated with adverse cardiovascular outcomes through behavioral, biological, and psychological pathways [20]. Behaviorally, our observed clustering of smoking and physical inactivity among lonely MI patients suggests that patients may adopt maladaptive coping mechanisms that could exacerbate cardiovascular risk.

Previous studies have proposed behavioral, psychological, and biological mechanisms that may contribute to the association between loneliness and cardiovascular health [20]. For example, loneliness has been linked to neuroendocrine, cardiovascular, and inflammatory stress responses [21]. However, these mechanisms were not assessed in the present study and therefore cannot be inferred from our findings. Our findings are also consistent with emerging international evidence linking loneliness and social isolation with adverse cardiovascular outcomes [22]. For example, Liang et al. [23] demonstrated that social isolation and loneliness were independently associated with an increased risk of incident heart failure in a large population‐based cohort study. In another study, the same authors reported that loneliness and social isolation were associated with a higher risk of major adverse cardiovascular events among individuals with type 2 diabetes mellitus. These findings collectively support the growing recognition of psychosocial factors as important correlates of cardiovascular health across different populations and clinical settings [5, 11]. A meta‐analysis found that both actual and perceived loneliness significantly increased the risk of early mortality by up to 32%, exhibiting effects comparable to those of other recognized risk factors, regardless of sex or region [24]. In the Saudi sociocultural setting, these mechanisms may be intensified by rapid modernization, urban migration, and changing family structures, which can weaken traditional social support networks. For example, younger adults may experience greater loneliness despite living with extended family due to digital isolation and changing social norms. Thus, while the biological and behavioral mechanisms mirror global patterns, the social context may amplify their impact, underscoring the need for culturally contextualized prevention strategies. Importantly, our interpretations beyond the observed statistical associations are presented as theoretical implications rather than data‐derived conclusions.

Interestingly, we found that younger MI patients (≤ 50 years) were almost twice as likely to report high loneliness as older adults. This is somewhat unexpected, because older people are believed to be the most socially isolated. Digital isolation may represent one possible explanation for the greater loneliness observed among younger patients; however, this factor was not assessed in the present study. Future longitudinal studies should examine its potential contribution to loneliness in younger cardiac populations.

Additionally, physical inactivity and smoking were associated with higher odds of loneliness in our study. Given the cross‐sectional design, the direction of these associations cannot be determined. Potential behavioral explanations for these findings were not directly assessed and require investigation in longitudinal studies.

Our analysis revealed that multiple comorbidities substantially increased the likelihood of loneliness, tripling the risk among cardiac patients who were socially isolated compared to others. In particular, individuals with CKD demonstrated higher odds of loneliness, which may reflect the psychosocial burden of chronic illness, reduced mobility, and frequent medical dependency. These findings emphasize the importance of multidisciplinary care, including psychological and social support, for cardiac patients with multiple comorbidities, particularly CKD and HTN. Conversely, higher physical activity levels and appropriate diabetes management appeared to have a modest association with lower odds of loneliness, potentially due to improved functional capacity and engagement with healthcare systems. Physical activity has been linked to loneliness in different studies. For example, a cross‐sectional study in European countries found that physically inactive people were highly socially isolated [25]. Another study showed a dramatic increase in loneliness scores, along with a decrease in physical function, among older adults, particularly with increasing age [12]. These findings suggest that maintaining physical activity and adhering to chronic disease management may mitigate feelings of isolation among cardiac patients. Additionally, the observed inverse association between obesity and loneliness among cardiac patients should be interpreted with caution. Given the subgroup sample size and the number of included covariates, this finding may reflect residual confounding, sample‐specific variation, or model instability rather than a true association relationship. Therefore, the findings of this subgroup analysis should be considered exploratory and hypothesis‐generating rather than confirmatory and require validation in larger longitudinal studies. Beyond individual health status, other social determinants—including low educational level, unemployment status, and low household income—were significantly associated with loneliness in bivariate analyses. These results align with the existing literature, showing that socioeconomic status is a key determinant of both social and cardiovascular health outcomes [20]. Individuals with limited financial and social resources may encounter challenges in accessing healthcare and community support, which could exacerbate their risk of isolation and cardiovascular issues. In Saudi Arabia, sociocultural norms, resource limitations, and environmental factors significantly affect lifestyle behaviors and the accessibility of healthcare services [14]. Additionally, low education level and various sociocultural norms result in the discouragement of healthy behaviors in cardiac patients in Saudi Arabia following a cardiac event [13].

This study’s findings shed some light on a much‐neglected impact of CVD that needs more attention, especially in Saudi Arabia. For instance, one study explored the implementation of lifestyle interventions in patients with CVD, identifying the main obstacles as limited time, a lack of training, low patient motivation, and a lack of institutional support, while highlighting the need for culturally appropriate education and systemic improvements to enhance cardiac care [13]. The current findings extend the understanding of loneliness as a psychosocial associated factor for CVD in the Saudi Arabian context, where cultural and social structures differ from Western societies. Unlike most Western studies, which predominantly focus on elderly or postmenopausal populations, our results demonstrate a significant burden of loneliness among younger adults with MI. This highlights the need to broaden preventive strategies beyond focusing on the elderly to include younger cardiac patients who may be socially or occupationally vulnerable. The observed association between loneliness and comorbidities, such as CKD and hypertension, further underscores the necessity of integrating psychosocial screening and counseling into healthcare systems, especially in cardiology settings and cardiac rehabilitation programs. These findings may guide clinicians to adopt a more holistic approach, combining medical management with social support interventions. The differences from prior international studies may reflect variations in family structure, lifestyle, and societal expectations in Saudi Arabia, which can influence social connectivity and emotional well‐being after cardiac events. These findings support further investigation of culturally appropriate approaches such as digital health apps to address loneliness among cardiac patients. However, whether psychosocial or social‐connectivity interventions improve cardiovascular outcomes requires evaluation in prospective interventional studies. These strategies should target younger as well as older adults, patients with comorbidities, and patients experiencing socioeconomic difficulties. Future research should use longitudinal and interventional designs to investigate the causal pathways linking loneliness and CVD in Saudi Arabia. In particular, studies integrating biomarkers of stress, inflammation, and autonomic dysfunction may clarify the biological mechanisms underlying this association. Additionally, qualitative studies are needed to understand the sociocultural perceptions of loneliness among different age and gender groups in the Saudi context.

### 4.1. Limitations

Despite using both bivariate and multivariable regression models, this study was limited by reliance on self‐reported data (social desirability bias), community‐based samples, and a relatively small sample size due to patients denying living in loneliness, particularly given the sensitive nature of loneliness‐related questions. In addition, it is a questionnaire‐based study, and such studies have well‐known weaknesses. Although the calculated target sample size for MI patients was not fully achieved, the study still identified several statistically significant associations. Nevertheless, the reduced sample size may have limited statistical power and the generalizability of the findings. Additionally, some subgroup analyses may have been affected by limited sample size relative to the number of included predictors, which may increase the risk of residual confounding or model overfitting.

### 4.2. Conclusion

This study revealed that approximately one‐third of the Saudi Arabian participants experienced moderate to high levels of loneliness, indicating loneliness may represent an emerging public health concern. Our results suggest that loneliness extends beyond psychological concerns. Loneliness was significantly associated with a history of MI and with key behavioral and clinical factors such as smoking, physical inactivity, and comorbidities. Although these associations persisted after adjustment for confounders, the cross‐sectional design of this study precludes causal inference. Therefore, loneliness should be viewed as a potential psychosocial correlate, rather than as an independent predictor, of CVD. These findings underscore the importance of incorporating social well‐being into cardiovascular prevention and rehabilitation strategies and highlight the need for longitudinal studies to further explore the directionality and mechanisms of these associations in the Saudi population.

**Nomenclature**
CVD: Cardiovascular disease
MI: Myocardial infarction
DALYs: Disability‐adjusted life years
SDI: Sociodemographic index
BMI: Body mass index
CKD: Chronic kidney diseases
HTN: Hypertension
COPD: Chronic obstructive pulmonary disease

## Author Contributions

Hessah Alshammari conceptualized the study, performed the statistical analysis, contributed to the study design, supervised data collection, reviewed the manuscript critically, and led the manuscript writing.

Norah Alshafi, Tharaa Alhowaish, Lina Almohsen, and Dena Alsuhaibani performed data collection and analysis.

Sarah Mazi contributed to the literature review and helped in editing the final draft of the manuscript. Hassan Alabdullatif created the idea and wrote the research proposal. Thamir Al‐khlaiwi coordinated the research activities, ensured ethical compliance, participated in data interpretation, contributed to drafting and revising the manuscript, and provided overall supervision.

## Funding

This research was funded by King Saud University, Riyadh, Saudi Arabia, through the Ongoing Research Funding Program (Grant No. ORF‐2026‐1974).

## Disclosure

The funding body had no role in the study design; collection, analysis, or interpretation of data; writing of the manuscript; or the decision to submit the manuscript for publication.

## Ethics Statement

This study was approved by the Institutional Review Board of the College of Medicine, King Saud University (approval number: Research Project No. E‐24‐8857). All participants provided written informed consent for publication of anonymized data before participating in the study.

## Consent

Please see the Ethics Statement.

## Conflicts of Interest

The authors declare no conflicts of interest.

## Supporting Information

Additional supporting information can be found online in the Supporting Information section.

## Supporting information


**Supporting Information** The supporting information provides additional information supporting the findings presented in the main manuscript and includes Table S1, which presents a descriptive analysis of the sociodemographic, lifestyle, and loneliness‐related characteristics of the participants included in the study. The table summarizes the participants’ sex, age group, body mass index category, marital status, educational level, employment status, monthly household income, smoking status and smoking intensity, alcohol consumption, physical activity level, and self‐rated De Jong Gierveld Loneliness Scale score categories. Frequencies and percentages are provided for each characteristic. Supporting Table S2 presents a descriptive analysis of the patients’ medical and cardiac history. The table summarizes the prevalence and duration of previous MI, the type of diagnosed MI, IHD classification, the number of previous MIs, recurrent cardiac events and complications, comorbidities, and cardiac‐related treatments received by the study participants. Supporting Table S3A presents the bivariate analysis of sociodemographic, lifestyle, and clinical characteristics associated with a high loneliness score, defined as a loneliness score ≥ 3. The analysis compares participants with low loneliness scores and those with high loneliness scores using chi‐square tests of independence. Variables examined include sex, age group, marital status, educational level, employment status, household monthly income, smoking status and smoking level, alcohol consumption, physical activity level, and body mass index. Supporting Table S3B presents the bivariate analysis of medical history, cardiac history, comorbidities, and cardiac treatments in relation to high loneliness, defined as a loneliness score ≥ 3. Participants with and without high loneliness scores were compared using chi‐square tests. The analysis included previous MI, time since the last MI, MI type, IHD, number of previous MIs, recurrent cardiac events and complications, individual comorbidities, and cardiac treatments received.

## Data Availability

The datasets generated and analyzed during the current study are not publicly available because of ethical and institutional restrictions related to participant confidentiality.
