# Supplementary material for: The Association Between Myocardial Infarction and Loneliness Among Adults in the Saudi Population: A Comparative Analytical Study
Source: Cardiol Res Pract. 2026 Oct 23;2026:6360092. doi: 10.1155/crp/6360092 (PMC13691624; doi:10.1155/crp/6360092)
Supplement: Supplementary file 1 [file CRP-2026-6360092-s001.zip › Table S2.pdf]

Table S2: Descriptive analysis of the patients medical and cardiac history.		
	Frequency	Percentage
Medical history of Myocardial Infarction		
No	473	75.8
Yes	151	24.2
Duration (years) since last Myodardial		
1-2 years	48	7.7
3-4 years	61	9.8
>= 5 years	42	6.7
Not affected	473	75.8
Type of diagnosed Myocardial Infarction		
Not affected	473	75.8
MI related to CABG	3	0.5
MI secondary to ischemic	48	7.7
MI secondary to restenosis after PCI	7	1.1
MI secondary to stent thrombosis	3	0.5
MI within 48 hours of PCI	1	0.2
Spontanoeus MI	89	14.3
Ischemic Heart Disease		
Unaffected	473	75.8
NSTEMI	28	4.5
Stable angina	12	1.9
STEMI	103	16.5
Unstable angina	8	1.3
Number of affected Myocardial		
None	473	75.8
One MI's	117	18.8
Two MI's	25	4
Three MI's	9	1.4
Recurrent cardiac events and complications (Dhysrhythmias, Low EF,..)		
No	599	96
Yes	25	4
Comorbidity, n=350 (56.1%)		
Hypertension	166	47.7
Diabetes	229	65.4
Dyslipidemia	231	66
Chronic Kideny Dysfunction disease	30	8.6
COPD	20	5.7
PAD	26	7.4
CVD	30	8.6
Physical Disability	4	1.1
Hypothyroidims	7	2
Benign Prostatic Hypertrophy	7	2
Another disease/multi-comorbidity	37	10.6

Cardiac Received Treatments, n=321.		
Antiplatelets	172	53.6
Beta Blockers	132	41.1
ACE inhibitors	135	42.1
Statins	258	80.4
Nitrates	9	2.8
Anticoagulants	26	8.1
Hypoglycemic agents	174	54.2
Benzodiazepines	1	0.3
Anti-Depressants	11	3.4
Opiods	4	1.2
Diuretic agents	37	11.5
Other medications	46	14.3
