# Supplementary material for: The Association Between Myocardial Infarction and Loneliness Among Adults in the Saudi Population: A Comparative Analytical Study
Source: Cardiol Res Pract. 2026 Oct 23;2026:6360092. doi: 10.1155/crp/6360092 (PMC13691624; doi:10.1155/crp/6360092)
Supplement: Supplementary file 1 [file CRP-2026-6360092-s001.zip › Table S3A.pdf]

Table S3A: Bivariate analysis of individuals' risk of experiencing high loneliness (loneliness score ≥3)				
	High loneliness score			
	No	Yes	test statisticχ2	p-value
**Sex**				
Male	282 (63.1)	121 (68.4)	χ2(1)=1.54	0.214
Female	165 (36.9)	56 (31.6)		
**Age group**				
<30 years	64 (14.3)	26 (14.7)	χ2(3)=6.12	0.106
30-50 years	161 (36)	46 (26)		
51-70 years	185 (41.4)	87 (49.2)		
>70 years	37 (8.3)	18 (10.3)		
**Marital state**				
Never married	63 (14.1)	29 (16.4)	χ2 (2)=2.68	0.261
Divorced/widowed	33 (7.4)	19 (10.7)		
Married	351 (78.5)	129 (72.9)		
**Educational Level**				
Less than High school	38 (8.5)	33 (18.6)	χ2(1)=13.84	0.003
High school	102 (22.8)	35 (19.8)		
University degree	222 (49.7)	84 (47.5)		
Postgraduate studies	85 (19)	25 (14.1)		
**Employment status**				
No	174 (38.9)	91 (51.4)	χ2(1)=13.4	0.001
Part-time employed	14 (3.1)	11 (6.2)		
Full-time employed	259 (57.9)	75 (42.4)		
**Household monthly income**				
Low income	111 (24.8)	51 (28.8)	χ2(2)=7.12	0.028
Intermediate income	212 (47.4)	95 (53.7)		
High Income	124 (27.7)	31 (17.5)		
**Smoking status**				
Non-smoker	329 (73.6)	105 (59.3)	χ2(1)=12.21	<0.001
Smoker	118 (26.4)	72 (40.7)		
**Smoking Level**				
Non-smoker	329 (73.6)	105 (59.3)	χ2(3)=19.26	<0.001
<10 cigarettes per day	7 (1.6)	12 (6.8)		
10-19 cigarettes per day	46 (10.3)	22 (12.4)		
>20 cigarettes per day	65 (14.5)	38 (21.5)		
**Alcohol consumption**				
No	444 (99.3)	173 (97.7)	χ2(1)=1.63	0.202
Yes	3 (0.7)	4 (2.5)		

**Physical activity level**				
Inactive	191 (42.7)	75 (42.4)	χ2(1)=4.70	0.095
30-90 Minutes per week	160 (35.8)	76 (42.9)		
>90 minutes per week	96 (21.5)	26 (14.7)		
**Body mass index**				
Underweight	4 (0.9)	0 (0)	χ2(5)=7.62	0.178
Normal	104 (23.3)	50 (28.2)		
Overweight	175 (39.1)	62 (35)		
Obesity class I	93 (20.8)	45 (25.4)		
Obesity class II	52 (11.6)	15 (8.5)		
Obesity class III	19 (4.3)	5 (2.8)		
