# Supplementary material for: The Association Between Myocardial Infarction and Loneliness Among Adults in the Saudi Population: A Comparative Analytical Study
Source: Cardiol Res Pract. 2026 Oct 23;2026:6360092. doi: 10.1155/crp/6360092 (PMC13691624; doi:10.1155/crp/6360092)
Supplement: Supplementary file 1 [file CRP-2026-6360092-s001.zip › Table S3B.pdf]

**Table S3B: Bivariate analysis of risk of experiencing high loneliness (loneliness score ≥3 Points)**				
	High loneliness score			
	No	Yes	test statistiχ2	p-value
**Medical history of myocardial infarction (MI).**				
No	379 (84.8)	94 (53.1)	χ2(1)=69.4	<0.001
Yes	68 (15.2)	83 (46.9)		
**Duration (years) since last MI**				
1-2 years	20 (4.5)	28 (15.8)	χ2(3)=71.1	<0.001
3-4 years	31 (6.9)	30 (16.9)		
>= 5 years	17 (3.8)	25 (14.1)		
Not affected	379 (84.8)	94 (53.1)		
**Type of diagnosed MI**				
Not affected	379 (84.4)	94 (53.1)	χ2(6)=72.64	<0.001
MI related to CABG	1 (0.2)	2 (1.1)		
MI secondary to ischemic	19 (4.3)	29 (16.4)		
MI secondary to restenosis after PCI	2 (0.4)	5 (2.8)		
MI secondary to stent thrombosis	3 (0.7)			
MI within 48 hours of PCI		1 (0.6)		
Spontaneous MI	43 (9.6)	46 (26)		
**Ischemic heart disease**				
Unaffected	379 (84.8)	94 (53.1)	χ2(4)=66.98	<0.001
NSTEMI	13 (2.9)	15 (8.5)		
Stable angina	3 (0.7)	9 (5.1)		
STEMI	48 (10.7)	55 (31.1)		
Unstable angina	4 (0.9)	4 (2.3)		
**Number of MIs**				
None	379 (84.4)	94 (53.1)	χ2(3)=65.7	<0.001
One MI	52 (11.6)	65 (36.7)		
Two MIs	13 (2.9)	12 (6.8)		

Three MIs	3 (0.7)	6 (3.4)		
**Recurrent cardiac events and complications.**				
No	437 (97.8)	162 (91.5)	χ2(1)=	122.83
Yes	10 (2.2)	15 (8.5)		
Comorbidity				
Dyslipidemia	167 (37.4)	64 (36.2)	χ2(1)=0.10	0.779
Diabetes	155 (34.7)	74 (41.8)	χ2(1)=2.77	0.096
Hypertension	98 (21.9)	68 (38.4)	χ2(1)=17.66	<0.001
Other disease/multi-comorbidity	13 (2.9)	24 (13.6)	χ2(1)=25.8	<0.001
Chronic kidney dysfunction	16 (3.6)	14 (7.9)	χ2(1)=5.19	0.023
CVD	16 (3.6)	14 (7.9)	χ2(1)=5.19	0.023
PAD	17 (3.8)	9 (5.1)	χ2(1)=0.522	0.47
Multiple comorbidities	14 (3.1)	6 (3.4)	χ2(1)=0.027	0.869
Hypothyroidism	2 (0.4)	5 (2.8)	χ2(1)=4.39	0.034
Benign prostatic hypertrophy		7 (4)	χ2(1)=14.50	<0.001
Physical disability	3 (0.7)	1 (0.6)	χ2(1)=0.022	0.881
**Cardiac treatment received**				
Statins	161 (36)	80 (45.2)	χ2(1)=18.5	<0.001
Hypoglycemic agents	120 (26.8)	54 (30.5)	χ2(1)=0.85	0.358
Antiplatelets	96 (21.5)	76 (42.9)	χ2(1)=29.25	<0.001
ACE inhibitors	69 (15.4)	66 (37.3)	χ2(1)=35.7	<0.001
Beta Blockers	66 (14.8)	66 (37.3)	χ2(1)=38.6	<0.001
Other medications	20 (4.5)	26 (14.7)	χ2(1)=19.4	<0.001
Diuretic agents	14 (3.1)	23 (13)	χ2(1)=22.1	<0.001
Anticoagulants	12 (2.7)	14 (7.9)	χ2(1)=8.67	0.003
Antidepressants	9 (2)	2 (1.1)	χ2(1)=0.18	0.676
Nitrates	4 (0.9)	5 (2.8)	χ2(1)=3.32	0.068
Opioids	3 (0.7)	1 (0.6)	χ2(1)=0.0001	1
Benzodiazepines		1 (1.6)	χ2(1)=0.231	0.112
