# Supplementary material for: The Association Between Myocardial Infarction and Loneliness Among Adults in the Saudi Population: A Comparative Analytical Study
Source: Cardiol Res Pract. 2026 Oct 23;2026:6360092. doi: 10.1155/crp/6360092 (PMC13691624; doi:10.1155/crp/6360092)
Supplement: Supplementary file 1 [file CRP-2026-6360092-s001.zip › Supplementary (1).docx]

| **Table S1: Descriptive analysis of the participents’ sociodemographic characteristics. N=624** | | |
| --- | --- | --- |
|  | Frequency | Percentage |
| Sex |  |  |
| Male | 403 | 64.6 |
| Female | 221 | 35.4 |
| Age group |  |  |
| <30 years | 90 | 14.4 |
| 30-50 years | 207 | 33.2 |
| 51-70 years | 272 | 43.6 |
| >70 years | 55 | 8.8 |
| Body Mass Index Level |  |  |
| Underweight | 4 | 0.6 |
| Normal | 154 | 24.7 |
| Overweight | 237 | 38 |
| Obesity class I | 138 | 22.1 |
| Obesity class II | 67 | 10.7 |
| Obesity class II | 24 | 3.8 |
| Marital state |  |  |
| Never married | 92 | 14.7 |
| Divorced/widowed | 52 | 8.3 |
| Married | 480 | 76.9 |
| Educational Level |  |  |
| Less than High school | 71 | 11.4 |
| High school | 137 | 22 |
| University degree | 306 | 49 |
| Post-graduate studies | 110 | 17.6 |
| Employment | 624 | 100 |
| Not employed | 265 | 42.5 |
| Part-time employed | 25 | 4 |
| Full-time employed | 334 | 53.5 |
| Household montly income |  |  |
| Low income | 162 | 26 |
| Intermediate income | 307 | 49.2 |
| High Income | 155 | 24.8 |
| Smoking status |  |  |
| Non-smoker | 434 | 69.6 |
| Smoker | 190 | 30.4 |
| Smoking Level |  |  |
| Non-smoker | 434 | 69.6 |
| <10 cigarettes per day | 19 | 3 |
| 10-19 cigarettes per day | 68 | 10.9 |
| >=20 cigarettes per day | 103 | 16.5 |
| Alcohol consuming |  |  |
| No | 617 | 98.9 |
| Yes | 7 | 1.1 |
| Physical activity Level |  |  |
| Inactive | 266 | 42.6 |
| 30-90 Minutes per week | 236 | 37.8 |
| >90 minutes per week | 122 | 19.6 |
| Self-rated DeJong Gierveld Loneliness Scale score levels | |  |
| 0=Very low | 224 | 35.9 |
| 1=Low | 123 | 19.7 |
| 2=Mild | 100 | 16 |
| 3=Moderate | 94 | 15.1 |
| 4=High | 35 | 5.6 |
| 5=Very high | 26 | 4.2 |
| 6= extreme | 22 | 3.5 |

| **Table S2: Descriptive analysis of the patients medical and cardiac history.** | | |
| --- | --- | --- |
|  | Frequency | Percentage |
| Medical history of Myocardial Infarction (MI). |  |  |
| No | 473 | 75.8 |
| Yes | 151 | 24.2 |
| Duration ( years) since last Myodardial Infarction |  |  |
| 1-2 years | 48 | 7.7 |
| 3-4 years | 61 | 9.8 |
| >= 5 years | 42 | 6.7 |
| Not affected | 473 | 75.8 |
| Type of diagnosed Myocardial Infarction |  |  |
| Not affected | 473 | 75.8 |
| MI related to CABG | 3 | 0.5 |
| MI secondary to ischemic | 48 | 7.7 |
| MI secondary to restenosis after PCI | 7 | 1.1 |
| MI secondary to stent thrombosis | 3 | 0.5 |
| MI within 48 hours of PCI | 1 | 0.2 |
| Spontanoeus MI | 89 | 14.3 |
| Ischemic Heart Disease |  |  |
| Unaffected | 473 | 75.8 |
| NSTEMI | 28 | 4.5 |
| Stable angina | 12 | 1.9 |
| STEMI | 103 | 16.5 |
| Unstable angina | 8 | 1.3 |
| Number of affected Myocardial infarctions |  |  |
| None | 473 | 75.8 |
| One MI's | 117 | 18.8 |
| Two MI's | 25 | 4 |
| Three MI's | 9 | 1.4 |
| Recurrent cardiac events and complications (Dhysrhythmias, Low EF,..) | | |
| No | 599 | 96 |
| Yes | 25 | 4 |
| Comorbidity, n=350 (56.1%) |  |  |
| Hypertension | 166 | 47.7 |
| Diabetes | 229 | 65.4 |
| Dyslipidemia | 231 | 66 |
| Chronic Kideny Dysfunction disease | 30 | 8.6 |
| COPD | 20 | 5.7 |
| PAD | 26 | 7.4 |
| CVD | 30 | 8.6 |
| Physical Disability | 4 | 1.1 |
| Hypothyroidims | 7 | 2 |
| Benign Prostatic Hypertrophy | 7 | 2 |
| Another disease/multi-comorbidity | 37 | 10.6 |
| Cardiac Received Treatments, n=321. |  |  |
| Antiplatelets | 172 | 53.6 |
| Beta Blockers | 132 | 41.1 |
| ACE inhibitors | 135 | 42.1 |
| Statins | 258 | 80.4 |
| Nitrates | 9 | 2.8 |
| Anticoagulants | 26 | 8.1 |
| Hypoglycemic agents | 174 | 54.2 |
| Benzodiazepines | 1 | 0.3 |
| Anti-Depressants | 11 | 3.4 |
| Opiods | 4 | 1.2 |
| Diuretic agents | 37 | 11.5 |
| Other medications | 46 | 14.3 |

| **Table S3A: Bivariate analysis of individuals’ risk of experiencing high loneliness (loneliness score ≥3) (N=619).** | | | | |
| --- | --- | --- | --- | --- |
|  | High loneliness score |  |  | |
|  | No | Yes | test statistiχ2 | p-value |
| **Sex** |  |  |  |  |
| Male | 282 (63.1) | 121 (68.4) | χ2(1)=1.54 | 0.214 |
| Female | 165 (36.9) | 56 (31.6) |  |  |
| **Age group** |  |  |  |  |
| <30 years | 64 (14.3) | 26 (14.7) | χ2(3)=6.12 | 0.106 |
| 30-50 years | 161 (36) | 46 (26) |  |  |
| 51-70 years | 185 (41.4) | 87 (49.2) |  |  |
| >70 years | 37 (8.3) | 18 (10.3) |  |  |
| **Marital state** |  |  |  |  |
| Never married | 63 (14.1) | 29 (16.4) | χ2 (2)=2.68 | 0.261 |
| Divorced/widowed | 33 (7.4) | 19 (10.7) |  |  |
| Married | 351 (78.5) | 129 (72.9) |  |  |
| **Educational Level** |  |  |  |  |
| Less than High school | 38 (8.5) | 33 (18.6) | χ2(1)=13.84 | 0.003 |
| High school | 102 (22.8) | 35 (19.8) |  |  |
| University degree | 222 (49.7) | 84 (47.5) |  |  |
| Postgraduate studies | 85 (19) | 25 (14.1) |  |  |
| **Employment status** |  |  |  |  |
| No | 174 (38.9) | 91 (51.4) | χ2(1)=13.4 | 0.001 |
| Part-time employed | 14 (3.1) | 11 (6.2) |  |  |
| Full-time employed | 259 (57.9) | 75 (42.4) |  |  |
| **Household monthly income** |  |  |  |  |
| Low income | 111 (24.8) | 51 (28.8) | χ2(2)=7.12 | 0.028 |
| Intermediate income | 212 (47.4) | 95 (53.7) |  |  |
| High Income | 124 (27.7) | 31 (17.5) |  |  |
| **Smoking status** |  |  |  |  |
| Non-smoker | 329 (73.6) | 105 (59.3) | χ2(1)=12.21 | <0.001 |
| Smoker | 118 (26.4) | 72 (40.7) |  |  |
| **Smoking Level** |  |  |  |  |
| Non-smoker | 329 (73.6) | 105 (59.3) | χ2(3)=19.26 | <0.001 |
| <10 cigarettes per day | 7 (1.6) | 12 (6.8) |  |  |
| 10-19 cigarettes per day | 46 (10.3) | 22 (12.4) |  |  |
| ≥20 cigarettes per day | 65 (14.5) | 38 (21.5) |  |  |
| **Alcohol consumption** |  |  |  |  |
| No | 444 (99.3) | 173 (97.7) | χ2(1)=1.63 | 0.202 |
| Yes | 3 (0.7) | 4 (2.5) |  |  |
| **Physical activity level** |  |  |  |  |
| Inactive | 191 (42.7) | 75 (42.4) | χ2(1)=4.70 | 0.095 |
| 30-90 Minutes per week | 160 (35.8) | 76 (42.9) |  |  |
| >90 minutes per week | 96 (21.5) | 26 (14.7) |  |  |
| **Body mass index** |  |  |  |  |
| Underweight | 4 (0.9) | 0 (0) | χ2(5)=7.62 | 0.178 |
| Normal | 104 (23.3) | 50 (28.2) |  |  |
| Overweight | 175 (39.1) | 62 (35) |  |  |
| Obesity class I | 93 (20.8) | 45 (25.4) |  |  |
| Obesity class II | 52 (11.6) | 15 (8.5) |  |  |
| Obesity class III | 19 (4.3) | 5 (2.8) |  |  |

| **Table S3B: Bivariate analysis of risk of experiencing high loneliness (loneliness score ≥3 Points)** | | | | |
| --- | --- | --- | --- | --- |
|  | High loneliness score |  |  | |
|  | No | Yes | test statistiχ2 | p-value |
| **Medical history of myocardial infarction (MI).** |  |  | | |
| No | 379 (84.8) | 94 (53.1) | χ2(1)=69.4 | <0.001 |
| Yes | 68 (15.2) | 83 (46.9) |  |  |
| **Duration (years) since last MI** |  |  |  |  |
| 1-2 years | 20 (4.5) | 28 (15.8) | χ2(3)=71.1 | <0.001 |
| 3-4 years | 31 (6.9) | 30 (16.9) |  |  |
| >= 5 years | 17 (3.8) | 25 (14.1) |  |  |
| Not affected | 379 (84.8) | 94 (53.1) |  |  |
| **Type of diagnosed MI** |  |  |  | |
| Not affected | 379 (84.4) | 94 (53.1) | χ2(6)=72.64 | <0.001 |
| MI related to CABG | 1 (0.2) | 2 (1.1) |  |  |
| MI secondary to ischemic | 19 (4.3) | 29 (16.4) |  |  |
| MI secondary to restenosis after PCI | 2 (0.4) | 5 (2.8) |  |  |
| MI secondary to stent thrombosis | 3 (0.7) |  |  |  |
| MI within 48 hours of PCI |  | 1 (0.6) |  |  |
| Spontaneous MI | 43 (9.6) | 46 (26) |  |  |
| **Ischemic heart disease** |  |  |  |  |
| Unaffected | 379 (84.8) | 94 (53.1) | χ2(4)=66.98 | <0.001 |
| NSTEMI | 13 (2.9) | 15 (8.5) |  |  |
| Stable angina | 3 (0.7) | 9 (5.1) |  |  |
| STEMI | 48 (10.7) | 55 (31.1) |  |  |
| Unstable angina | 4 (0.9) | 4 (2.3) |  |  |
| **Number of MIs** |  |  |  | |
| None | 379 (84.4) | 94 (53.1) | χ2(3)=65.7 | <0.001 |
| One MI | 52 (11.6) | 65 (36.7) |  |  |
| Two MIs | 13 (2.9) | 12 (6.8) |  |  |
| Three MIs | 3 (0.7) | 6 (3.4) |  |  |
| **Recurrent cardiac events and complications.** | | | | |
| No | 437 (97.8) | 162 (91.5) | χ2(1)= | 122.83 |
| Yes | 10 (2.2) | 15 (8.5) |  |  |
| Comorbidity |  |  |  |  |
| Dyslipidemia | 167 (37.4) | 64 (36.2) | χ2(1)=0.10 | 0.779 |
| Diabetes | 155 (34.7) | 74 (41.8) | χ2(1)=2.77 | 0.096 |
| Hypertension | 98 (21.9) | 68 (38.4) | χ2(1)=17.66 | <0.001 |
| Other disease/multi-comorbidity | 13 (2.9) | 24 (13.6) | χ2(1)=25.8 | <0.001 |
| Chronic kidney dysfunction | 16 (3.6) | 14 (7.9) | χ2(1)=5.19 | 0.023 |
| CVD | 16 (3.6) | 14 (7.9) | χ2(1)=5.19 | 0.023 |
| PAD | 17 (3.8) | 9 (5.1) | χ2(1)=0.522 | 0.47 |
| Multiple comorbidities | 14 (3.1) | 6 (3.4) | χ2(1)=0.027 | 0.869 |
| Hypothyroidism | 2 (0.4) | 5 (2.8) | χ2(1)=4.39 | 0.034 |
| Benign prostatic hypertrophy |  | 7 (4) | χ2(1)=14.50 | <0.001 |
| Physical disability | 3 (0.7) | 1 (0.6) | χ2(1)=0.022 | 0.881 |
| **Cardiac treatment received** |  |  |  |  |
| Statins | 161 (36) | 80 (45.2) | χ2(1)=18.5 | <0.001 |
| Hypoglycemic agents | 120 (26.8) | 54 (30.5) | χ2(1)=0.85 | 0.358 |
| Antiplatelets | 96 (21.5) | 76 (42.9) | χ2(1)=29.25 | <0.001 |
| ACE inhibitors | 69 (15.4) | 66 (37.3) | χ2(1)=35.7 | <0.001 |
| Beta Blockers | 66 (14.8) | 66 (37.3) | χ2(1)=38.6 | <0.001 |
| Other medications | 20 (4.5) | 26 (14.7) | χ2(1)=19.4 | <0.001 |
| Diuretic agents | 14 (3.1) | 23 (13) | χ2(1)=22.1 | <0.001 |
| Anticoagulants | 12 (2.7) | 14 (7.9) | χ2(1)=8.67 | 0.003 |
| Antidepressants | 9 (2) | 2 (1.1) | χ2(1)=0.18 | 0.676 |
| Nitrates | 4 (0.9) | 5 (2.8) | χ2(1)=3.32 | 0.068 |
| Opioids | 3 (0.7) | 1 (0.6) | χ2(1)=0.0001 | 1 |
| Benzodiazepines |  | 1 (1.6) | χ2(1)=0.231 | 0.112 |
