# Supplementary material for: The Association Between Myocardial Infarction and Loneliness Among Adults in the Saudi Population: A Comparative Analytical Study
Source: Cardiol Res Pract. 2026 Oct 23;2026:6360092. doi: 10.1155/crp/6360092 (PMC13691624; doi:10.1155/crp/6360092)
Supplement: Supplementary file 1 [file CRP-2026-6360092-s001.zip › Table S1.pdf]

Table S1: Descriptive analysis of the participants' sociodemographic characteristics. N=624		
	Frequency	Percentage
Sex		
Male	403	64.6
Female	221	35.4
Age group		
<30 years	90	14.4
30-50 years	207	33.2
51-70 years	272	43.6
>70 years	55	8.8
Body Mass Index Level		
Underweight	4	0.6
Normal	154	24.7
Overweight	237	38
Obesity class I	138	22.1
Obesity class II	67	10.7
Obesity class II	24	3.8
Marital state		
Never married	92	14.7
Divorced/widowed	52	8.3
Married	480	76.9
Educational Level		
Less than High school	71	11.4
High school	137	22
University degree	306	49
Post-graduate studies	110	17.6
Employment	624	100
Not employed	265	42.5
Part-time employed	25	4
Full-time employed	334	53.5
Household montly income		
Low income	162	26
Intermediate income	307	49.2
High Income	155	24.8
Smoking status		
Non-smoker	434	69.6
Smoker	190	30.4
Smoking Level		
Non-smoker	434	69.6
<10 cigarettes per day	19	3
10-19 cigarettes per day	68	10.9
>=20 cigarettes per day	103	16.5
Alcohol consuming		
No	617	98.9

Yes	7	1.1
Physical activity Level		
Inactive	266	42.6
30-90 Minutes per week	236	37.8
>90 minutes per week	122	19.6
Self-rated DeJong Gierveld Loneliness Scale score levels		
0=Very low	224	35.9
1=Low	123	19.7
2=Mild	100	16
3=Moderate	94	15.1
4=High	35	5.6
5=Very high	26	4.2
6= extreme	22	3.5
